# Gastric Metastasis from Renal Cell Carcinoma, Clear Cell Type, Presenting with Gastrointestinal Bleeding

**DOI:** 10.1155/2017/5879374

**Published:** 2017-08-29

**Authors:** Mouhanna Abu Ghanimeh, Ayman Qasrawi, Omar Abughanimeh, Sakher Albadarin, John H. Helzberg

**Affiliations:** ^1^Internal Medicine Department, Division of Gastroenterology, Henry Ford Hospital, Detroit, MI, USA; ^2^Internal Medicine Department, University of Missouri-Kansas City School of Medicine, Kansas City, MO, USA; ^3^Division of Gastroenterology, University of Missouri-Kansas City School of Medicine, Kansas City, MO, USA; ^4^Saint Luke's Hospital of Kansas City, University of Missouri-Kansas City School of Medicine, Kansas City, MO, USA

## Abstract

Renal cell carcinoma (RCC) accounts for 80–85% of all primary renal neoplasms. Although RCC can metastasize to any organ, gastric metastases from RCC are exceedingly rare. A 67-year-old male presented with melena and acute blood loss anemia. The patient had a history of RCC that had been treated with a radical nephrectomy. He had a recent myocardial infarction and was receiving double antiplatelet therapy. After hemodynamic stabilization, esophagogastroduodenoscopy showed a polypoid mass in the gastric fundus. The mass was excised. Histological and immunohistochemical evaluation were consistent with clear cell RCC. The polypoid lesion is consistent with a late solitary metastasis.

## 1. Introduction

Renal cell carcinoma (RCC) is the most common cancer originating from the kidney [1]. Lungs, bones, liver, and brain are the most common sites of RCC metastasis [2, 3]. Uncommon metastatic sites, including the gastrointestinal tract [2–4], have also been reported. Gastric metastasis from RCC is rare [5, 6]. Gastric metastases are typically asymptomatic, single, and located in the gastric body or fundus [5, 6]. If they are symptomatic, then gastrointestinal bleeding and anemia are the most common presentations [5, 6]. RCC has the potential for late solitary metastasis. Isolated gastric metastasis from RCC can occur up to 20 years after radical nephrectomy [7]. Immunohistochemistry is useful and increasingly utilized in the diagnosis of RCC [8, 9]. The prognosis in patients with metastatic RCC is generally poor, with a five-year survival rate of 5–30% [10]. Treatment options include embolization and epinephrine injection for bleeding and endoscopic resection or surgery [11–16]. Surgical resection remains the best therapeutic option for a solitary gastric metastasis, resulting in significant survival prolongation in eligible patients [8].

## 2. Case Summary

A 67-year-old man presented with multiple episodes of melena. His past medical history involved polycystic kidney disease, live donor renal transplantation in 2002 with chronic immunosuppression, and metastatic left-sided RCC that had been treated with radical nephrectomy and the resection of a pulmonary metastasis in 2014. The patient had chronic kidney disease, stage 3, and a recent ST segment elevation myocardial infarction with percutaneous coronary intervention and drug eluting stent insertion. The patient was on 81 mg of aspirin daily and 90 mg of ticagrelor twice daily.

His vital signs on presentation were blood pressure of 121/82 mmHg, pulse of 105 bpm, and oral temperature of 97.7°F (36.5 C). On physical examination, the patient was pale and in mild distress. Abdominal and cardiopulmonary exams were unremarkable. Initial laboratory evaluation included a hemoglobin (Hb) level of 8.8 g/dl (normal: 13.5–17.5 g/dl), white blood cell (WBC) count of 11,300/cmm (normal: 4,000–11,000/cmm), platelet count of 344,000 cmm (normal: 150,000–450,000/cmm), serum creatinine level of 2.3 mg/dl (normal: 0.9–1.2 mg/dl), aspartate aminotransferase level of 27 units/L (normal: 15–46 units/L), alanine aminotransferase level of 14 units/L (normal: 13–69 units/L), alkaline phosphatase level of 117 units/L (normal: 42–140 units/L), and international normalized ratio of 1.2. The patient was admitted for stabilization and further evaluation of gastrointestinal bleeding.

**Figure 1:**
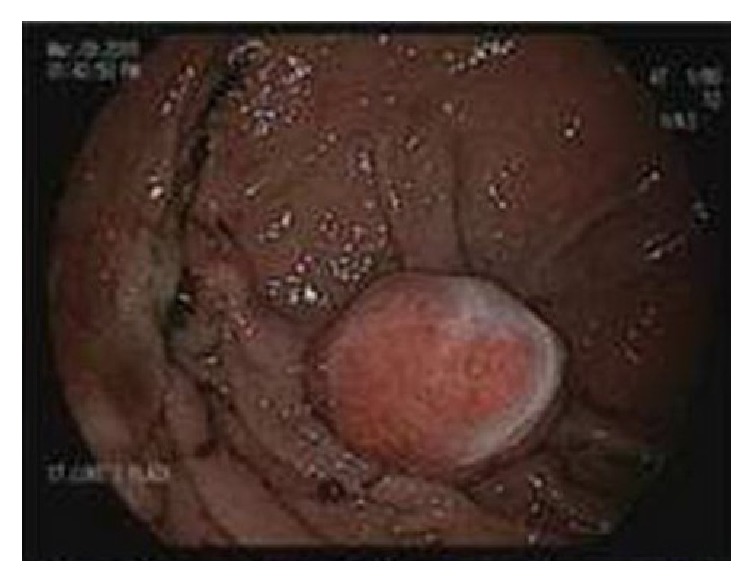
EGD showing a 2.5 to 3.0 cm polypoid mass in the gastric fundus.

The patient was intravenously given 80 mg pantoprazole, followed by 8 mg/hour continuous infusion. A total of 2 units of packed red blood cells were transfused. Aspirin and ticagrelor were initially held. On hospitalization day 1, the patient was hemodynamically stable and his Hb level increased to 9.9 g/dl after transfusion. The gastroenterology service proceeded with esophagogastroduodenoscopy (EGD). The EGD (Figure 1) showed a 2.5 to 3.0 cm polypoid mass in the gastric fundus. The polyp was completely removed with a polypectomy snare and cautery. Bleeding occurred after polyp removal, and hemostasis was achieved via local epinephrine injection and the application of two Cook hemostasis clips.

**Figure 2:**
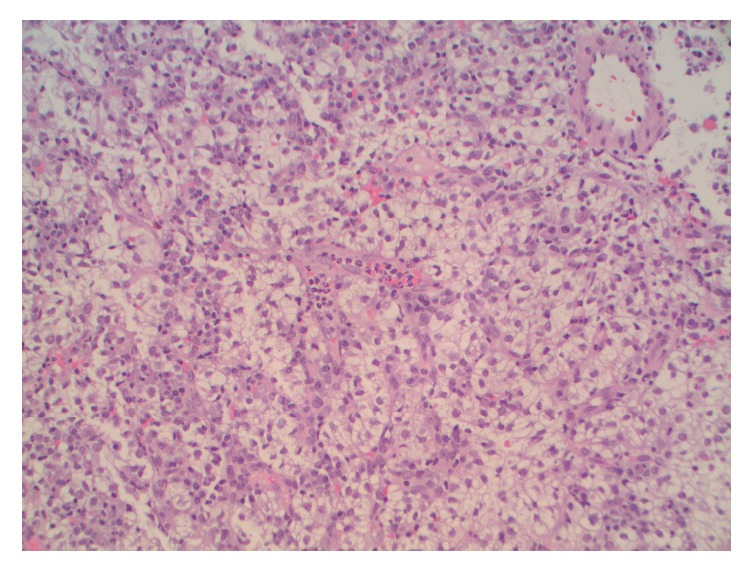
Histological evaluation including H&E staining, showing a tumor comprising nests and fascicles of cells with abundant clear cytoplasm and moderately pleomorphic nuclei with prominent eosinophilic nucleoli.

**Figure 3:**
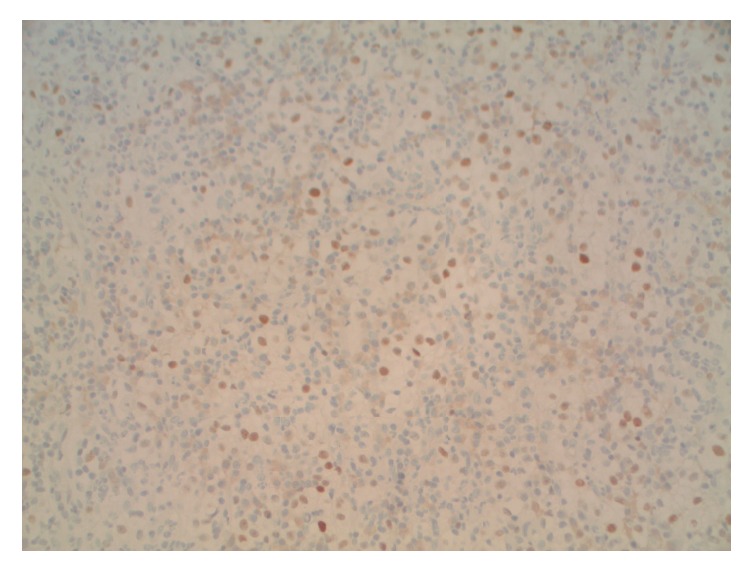
Positive immunohistochemical staining for PAX-2, consistent with clear cell RCC.

The histological examination (Figure 2) demonstrated a submucosal tumor comprising nests and fascicles of cells with abundant clear cytoplasm and moderately pleomorphic nuclei with prominent eosinophilic nucleoli. A background vascular network and acute and chronic inflammation were observed. Immunohistochemical staining (Figure 3) was positive for pan-keratin PAX2 and PAX8. Both the morphology and immune phenotypes were most consistent with metastatic clear cell RCC, comparable with the right lung lesion resected in 2014.

The patient was observed overnight in the intensive care unit. His Hb levels were unchanged, and he remained hemodynamically stable. Aspirin and ticagrelor treatments were resumed. The oncology service decided to follow him as an outpatient. Chemotherapy was not initiated with his recent gastrointestinal blood loss and myocardial infarction. He is following up now with the oncology and cardiology clinics and has been doing well about 1 year after his presentation.

## 3. Discussion

RCC is the most common cancer originating from the kidney. This cancer is responsible for 80 to 85% of all primary renal neoplasms and accounts for 3% of all adult malignancies [1]. RCC has an abundant blood supply and can metastasize to any organ [2, 3]. The most common sites of metastasis include the lungs, bones, liver, and brain [2, 3]. However, RCCs can also metastasize to unusual sites, including the pancreas, thyroid gland, adrenal gland, skeletal muscle, and skin [4]. Studies have reported that a metastasis is detected in approximately 30% of RCC patients on initial presentation [3].

Gastric metastases from RCC are exceedingly rare [5, 6]. Pollheimer et al. [5] reported 5 patients who developed gastric metastases from an Austrian database of 2,082 RCC patients. In one instance, an isolated gastric metastasis from RCC was reported 20 years after radical nephrectomy [7].  Table 1 summarizes the reported cases of gastric metastases from RCC in English literature.

Most RCC gastric metastases are located in the gastric body and fundus. Single tumors predominate over multiple tumors [6]. Histologically, these metastases are situated in the submucosa [3, 12]. Clear cell histology is the predominant form of RCC. The presence of clear cell morphology in any unknown lesion should prompt the pathologist to consider the possibility of metastatic RCC, even in the absence of a prior diagnosis [30]. Endoscopically, the metastasis typically appears as a polypoid submucosal-like tumor with a central depression.

In general, the outcome with metastatic RCC is poor with 5-year survival rates of 5–30% [10]. Patients with a single metastasis fare better than those with multiple metastases.

Immunohistochemistry, particularly for vimentin and PAX-2, is a useful adjunct in the diagnosis of RCC [8, 9]. Vimentin is an intermediate filament protein expressed in normal renal tissues [8], and PAX-2 is a transcription factor required for the development and proliferation of renal tubules [9]. Both proteins are expressed in 85% of metastatic clear cell RCCs [8, 9].

## Figures and Tables

**Table 1:** Reported cases of gastric metastases from RCC in English literature.

Case and reference	Age (years), sex	Gastrointestinal symptoms	Location	Gross appearance	Histology	Treatment
Sullivan et al. 1980 [17]	69, male	Bleeding	Antrum	Mass, single	Not specified	Antrectomy
Boruchowicz et al. 1995 [18]	48, male	Dysphagia	Fundus	Polypoid, single	Clear cell	Chemotherapy
Blake et al. 1995 [11]	63, male	Bleeding	Not specified	Not specified	Not specified	Embolization
Odori et al. 1998 [19]	58, male	Not specified	Not specified	Ulcerated, single	Clear cell	Total gastrectomy with regional lymph node dissection
Picchio et al. 2000 [12]	64, female	Bleeding	Body	Polyp, single	Clear cell	Subtotal gastrectomy
Mascarenhas et al. 2001 [20]	66, male	Bleeding	Body	Ulcerated, single	Clear cell	Partial gastrectomy
Kobayashi et al. 2004 [21]	78, male	Anemia	Lower one-third of stomach	Mass, single	Not specified	Gastrectomy
Kok Wee et al. 2004 [7]	60, male	Bleeding	Body	2 lesions, protruding and ulcerated	Clear cell	Endoscopic therapy
Lamb et al. 2005 [13]	69, male	Bleeding	Body	Mass, single	Clear cell	Embolization, octreotide
Riviello et al. 2006 [22]	68, male	Bleeding	Fundus	Mass, single	Clear cell	Total gastrectomy, chemotherapy
Pezzoli et al. 2007 [15]	78, male	Anemia	Body	Polyps, multiple	Clear cell	Endoscopic mucosal resection
Saidi and Remine 2007 [23]	Not specified	Bleeding	Body	Polyp, single	Clear cell	Wedge resection
Pollheimer et al. 2008 [5]	69, male	Epigastric pain, Nausea, vomiting	Body	Mass, single	Clear cell	Tamoxifen
Pollheimer et al. 2008 [5]	77, male	No symptoms	Antrum	Ulcerated, single	Clear cell	Interferon
Pollheimer et al. 2008 [5]	83, female	Bleeding	Antrum	Mass, multiple	Clear cell	Endoscopic therapy, interferon
Pollheimer et al. 2008 [5]	65, female	Bleeding	Not specified	Multiple	Clear cell	Endoscopic therapy
Pollheimer et al. 2008 [5]	69, male	Anemia, epigastric pain	Body	Multiple	Clear cell	Endoscopic therapy, sunitinib
Kibria et al. 2009 [24]	53, male	Bleeding	Fundus	Polypoid, single	Clear cell	None
Yamamoto et al. 2009 [8]	74, male	Bleeding	Body	Polypoid, single	Not specified	Wedge resection
Tiwari et al. 2010 [25]	58, female	Bleeding	Antrum	Polypoid, single	Clear cell	Subtotal gastrectomy
García-Campelo et al. 2010 [26]	75, male	No symptoms	Fundus and body	Polypoid, multiple	Not specified	Sunitinib
Sugasawa et al. 2010 [27]	69, male	Anemia	Fundus	Ulcerated, single	Clear cell	Wedge resection
Eslick and Kalantar 2011 [28]	65, male	Bleeding	Lower stomach	Polypoid, single	Clear cell	Polypectomy
Kim et al. 2012 [29]	79, male	Abdominal pain	Body	Erosive, single	Clear cell	Partial gastrectomy
Xu et al. 2012 [30]	60, male	Anemia	Body	Polyp, multiple	Clear cell	Polypectomy, sunitinib, sorafenib
Siriwardana et al. 2012 [31]	71, male	Anemia	Not specified	Polypoid, single	Clear cell	Endoscopic mucosal resection
Namikawa et al. 2012 [32]	65, male	Not specified	Body	Polypoid, single	Clear cell	Wedge resection
Rodrigues et al. 2012 [33]	45, female	Bleeding	Body	Ulcerated, single	Not specified	Sunitinib
Chibbar et al. 2013 [34]	69, female	Anemia	Body	Polypoid, single	Clear cell	Endoscopic mucosal resection
Rita et al. 2014 [6]	77, male	Bleeding, abdominal pain	Body	Polypoid, single	Clear cell	Endoscopic resection
Greenwald et al. 2014 [35]	62, male	No symptoms	Fundus	Mass, single	Clear cell	Partial gastrectomy
Costa et al. 2014 [36]	66, female	Anemia	Body	Ulcerated, single	Not specified	Laparoscopic wedge resection
Kumcu et al. 2014 [37]	59, male	Bleeding, weight loss	Body	Polypoid, single	Clear cell	Partial gastrectomy
Sakurai et al. 2014 [38]	62, male	Bleeding, anemia	Body	Mass, single	Clear cell	Partial gastrectomy
Forman et al. 2015 [39]	76, female	Bleeding, anemia	Cardia	Mass, single	Clear cell	Not specified
Kongnyuy et al. 2016 [40]	68, male	Anemia, bleeding	Fundus	Mass, single	Clear cell	Not specified
Our case 2016	67, male	Bleeding	Fundus	Polypoid, single	Clear cell	Polypectomy, plan for chemotherapy
